# Supervised and unsupervised language modelling in Chest X-Ray radiological reports

**DOI:** 10.1371/journal.pone.0229963

**Published:** 2020-03-10

**Authors:** Ignat Drozdov, Daniel Forbes, Benjamin Szubert, Mark Hall, Chris Carlin, David J. Lowe

**Affiliations:** 1 Bering Limited, London, United Kingdom; 2 Emergency Department, Queen Elizabeth University Hospital, Glasgow, Scotland; 3 Radiology Department, Queen Elizabeth University Hospital, Glasgow, Scotland; 4 Department of Respiratory Medicine. Queen Elizabeth University Hospital, Glasgow, Scotland; University of Central Florida (UCF), UNITED STATES

## Abstract

Chest radiography (CXR) is the most commonly used imaging modality and deep neural network (DNN) algorithms have shown promise in effective triage of normal and abnormal radiograms. Typically, DNNs require large quantities of expertly labelled training exemplars, which in clinical contexts is a major bottleneck to effective modelling, as both considerable clinical skill and time is required to produce high-quality ground truths. In this work we evaluate thirteen supervised classifiers using two large free-text corpora and demonstrate that bi-directional long short-term memory (BiLSTM) networks with attention mechanism effectively identify Normal, Abnormal, and Unclear CXR reports in internal (n = 965 manually-labelled reports, f1-score = 0.94) and external (n = 465 manually-labelled reports, f1-score = 0.90) testing sets using a relatively small number of expert-labelled training observations (n = 3,856 annotated reports). Furthermore, we introduce a general unsupervised approach that accurately distinguishes Normal and Abnormal CXR reports in a large unlabelled corpus. We anticipate that the results presented in this work can be used to automatically extract standardized clinical information from free-text CXR radiological reports, facilitating the training of clinical decision support systems for CXR triage.

## Introduction

Chest radiography (CXR) is the most commonly used imaging modality, with over two billion procedures performed annually [1]. There is a general consensus that an Artificial Intelligence (AI)-supported reporting of CXR images could be a valuable adjunct to imaging interpretation, providing substantial benefit in many clinical contexts, from improved workflow prioritization and clinical decision support to large-scale screening and global population health initiatives [2–4]. Indeed, deep learning algorithms have been successfully applied to detect heterogeneous thoracic disease [3, 5], triage normal and abnormal radiographs [2], and identify specific pathologies such as pulmonary tuberculosis [6], pneumonia [7], and lung cancer [8].

Deep learning models require large quantities of expertly labelled training exemplars [9] and the well-established computer science mantra “Garbage In, Garbage Out” holds especially true in clinical applications of AI [10]. Whilst the gold-standard of image annotation remains direct application of expert knowledge, the sheer size of the required datasets makes this endeavour impractical [11]. Therefore, Natural Language Processing (NLP) approaches offer an opportunity to automate the annotation of free-text reports [12]. For example, the Medical Language Extraction and Encoding (MedLEE) system relies on controlled vocabulary and grammatical rules to convert free text into a structured database [13]. PeFinder, an NLP system for pulmonary embolism classification, uses pre-defined lexical cues and context terms to achieve high sensitivity and positive predictive value [14]. Finally, NegEx, utilises hand-crafted regular expression rules to identify pertinent negatives from patient discharge summaries [15]. Nevertheless, applying text mining techniques to radiological reports, which may contain broken grammar and misspellings, poses a number of challenges due to extensive variability in linguistic ambiguity. Indeed, in the publicly-available ChestXray14 [16] imaging dataset, labels do not accurately reflect the visual content of the images, with positive predictive values of 10–30% lower than the values presented in the original documentation [11].

Neural network-driven modelling of radiological language has been proposed to supersede the hand-crafted rules and grammatical relations of the traditional rules-based algorithms [17]. Recently, a bi-directional long short-term memory (BiLSTM) network, which does not use any hand-engineered features, was demonstrated to perform favourably in a corpus of CXR reports (f1 = 0.87) [18]. Similarly, a supervised approach using a Recurrent Neural Network (RNN) with attention mechanism achieves high accuracy on expert-labelled CXR dataset (f1 = 0.93) [19]. Finally, Convolutional Neural Networks (CNNs) have been used to extract pulmonary embolism findings from thoracic computed tomography reports, outperforming state-of-the-art NLP systems (f1 = 0.94) [17].

Multi-label annotation of abnormal reports has been the primary aim of radiological language models [2, 4, 18, 19]. Nevertheless, practicalities of day-to-day clinical workflows suggest that the ability to identify ‘normal’ images and remove them from worklists would be anticipated to generate significant efficiency and cost savings [2, 20]. In addition, for clinicians reviewing images at the point of care, accurate triage of abnormal findings has potential safety, clinical outcome, and assistive (e.g. reduced cognitive overload) benefits [21, 22].

In this study we describe an approach to automatically extract standardized clinical information from free-text CXR radiological reports. More specifically, it is anticipated that accurate identification of Normal and Abnormal entities (irrespective of clinical sign or pathology) will facilitate training of AI-enabled triage systems at scale. We evaluate the utility of classical supervised machine learning techniques as well as state-of-the-art Long Short-Term Memory networks (LSTM) in the context of large corpora of free-text reports from Greater Glasgow and Clyde Health Board (n = 500,000) and the Beth Israel Deaconess Medical Center (MIMIC-CXR database [n = 227,835]) [23]. Additionally, we use ivis, an unsupervised Siamese Neural Network-based algorithm [24], which accurately classifies radiological reports and visualises document embeddings. Finally, we explore generalisability of machine learning techniques across European and North American radiological report corpora.

## Materials and methods

### Radiology reports and data preparation

Internal training, validation, and testing sets were produced using an in-house corpus of 500,000 deidentified CXR reports provided by NHS Greater Glasgow and Clyde (GGC) SafeHaven. NHS GGC is the largest health board in Europe and delivers health care for 1.1 million patients with seven cute hospital sites. The reports cover the period between January 2007 and January 2019. The repository consists of text typed or dictated by the clinicians after radiograph analysis and does not contain clinician or patient identifiable information such as names, addresses or dates of birth. The reports had a minimum of 1 word and maximum of 380 words, with an average of 33.2 words and standard deviation of 20.5 words. On average, there were 4.8 sentences per report. Prior to analysis, reports were converted to lower case and lemmatized. Numbers, punctuation marks, special characters, and words that occurred in fewer than three documents were discarded. The final vocabulary contained 9,598 words.

A random sample of 5,000 reports was selected from the corpus for the purpose of creating expert-labelled training, validation, and testing sets. The reports were manually labelled by a clinical fellow (DF) with special interest in Radiology. The annotation schema included three classes–Normal, Abnormal, and Uncertain. The decision on the labelling was guided by the Fleischner Society Glossary of Terms for Thoracic Imaging [25]. A report was deemed to be Normal if it was explicitly stated as such in the free-form report and if there were no reported medical or surgical paraphernalia (e.g. pacemaker, sutures). An Abnormal label was assigned to reports with at least one documented radiological sign or presence of medical or surgical paraphernalia. If a report was normal for the patient (e.g. hyperinflated lungs in a patient with known Chronic Obstructive Pulmonary Disease), the report was still categorised as Abnormal. In cases where insufficient clinical information was provided to reliably label a report as Normal or Abnormal, a label of Uncertain was assigned. Reports that were either blank or inconclusive (e.g. “see above”, “same as above”) were excluded from the labelling exercise. All reports were labelled using the open source text annotation tool Doccano [26]. The final labelled corpus consisted of 4,821 reports.

The external testing set was drawn from 227,835 radiographic studies recorded within the MIMIC-CXR database [23]. A random sample of 500 reports was selected from the corpus for the purpose of creating an expert-labelled testing set. Pre-processing and annotation were performed as above. Following exclusion of inconclusive reports (e.g. reported only as “as above”, “see above”), the final external testing corpus contained 465 reports. The reports had a minimum of 2 words and maximum of 118 words, with an average of 13.4 words and standard deviation of 16.2 words. On average, there were 2.9 sentences per report.

### Supervised report classification

Expert-annotated reports were used to train three types of supervised classifiers: non-neural (i.e. classical machine learning algorithms), LSTM-based, and attention-based models (Transformers).

#### Non-neural classifiers

**Fig 1:**
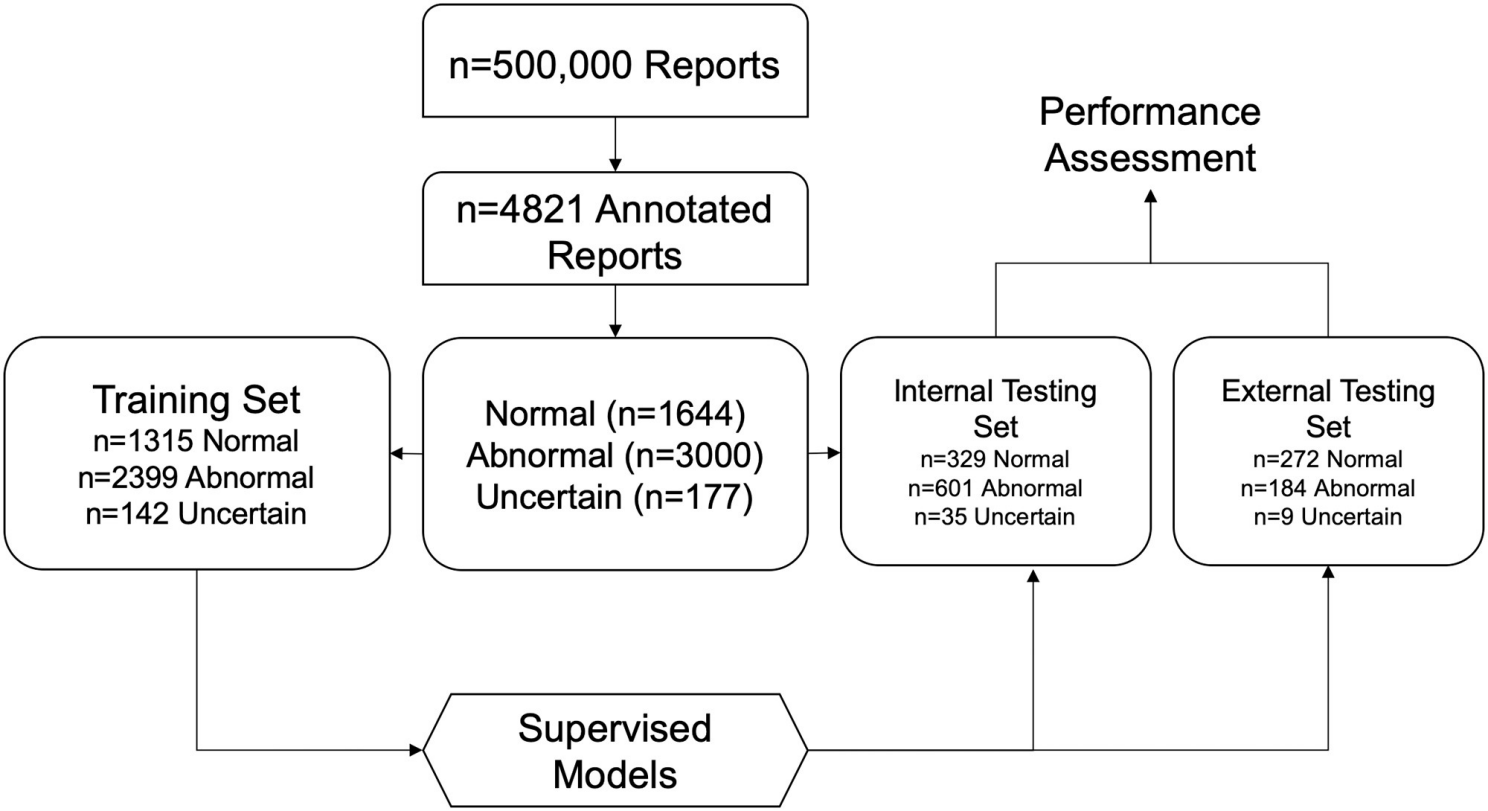
Flowchart showing supervised approach to radiology report classification.

The labelled corpus, consisting of Normal, Abnormal, and Unclear reports, was converted to term frequency-inverse document frequency (tf-idf) matrix and reduced to 100 dimensions using Singular Value Decomposition (SVD). Subsequently, the transformed matrix was randomised into training (80%) and testing sets (20%) using a stratified split (Fig 1). Five supervised machine learning algorithms were evaluated on tf-idf/SVD-transformed radiology reports–K-Nearest Neighbour Classifier (KNN), Logistic Regression (LR), Gaussian Naïve Bayes Classifier (NBC), Random Forest (RF), and Support Vector Machine (SVM). Each model’s hyperparameters were tuned on the training set using a Grid Search algorithm with stratified five-fold cross-validation. Hyperparameters that yielded the best macro-averaged f1 statistic across the five folds were retained for predictions on the independent testing set.

#### LSTM classifiers

The internal labelled corpus, consisting of Normal, Abnormal, and Unclear reports, was randomised into training (80%) and testing sets (20%) using stratified splits. Each report was then represented as a tokenised sequence of words. We limited the maximum length of the input sequence to 40, padding shorter sequences with zeros, whilst cropping longer sequences. Model inputs were mapped to an Embedding layer, which was initialised either by using either pre-trained fastText [27] weights or by drawing from a uniform distribution in the (−0.01, 0.01) range. The fastText model was trained on an unlabelled corpus of lemmatised and pre-processed free-text reports (n = 495,179, see above). Window size was set to three and embedding dimensionality was set to 50. Subsequently, a Bidirectional LSTM (BiLSTM) architecture [28] was implemented, with each LSTM layer consisting of 100 memory cells. The loss function was the categorical cross-entropy between the predicted probabilities of the report tags and the true tags.

Our BiLSTM model was also supplemented with an attention mechanism (BiLSTM-ATT) [19, 29], in which the BiLSTM layer is followed with an attention module. The attention module generates a predictive distribution over the LSTM encodings for each step by firstly calculating the dot-product of the latest hidden state and the previous states, and then using the SoftMax function [30]. Applying these scores to the previous hidden state vectors effectively samples the most useful input vectors dynamically by predicting which vectors are most important for the predictions. By enabling selective sampling of relevant information from all encoder states, the model is able to deal with long sequences of words and maintain global information about the input sentence. Finally, all models were trained for 20 epochs with batches of 32 sentences using the Adam optimiser with the learning rate set to 0.001. Training was terminated early if the validation loss did not improve for three consecutive epochs.

#### Transformer classifiers

The Transformer is a novel neural network architecture based solely on a self-attention mechanism [31]. Four Transformer models were trained on the internal training set—Bidirectional Encoder Representations from Transformers (BERT) [32], DistilBERT [33], XLNet [34], and RoBERTa [35]. Each Transformer model was initialised using pre-trained weights provided by the HuggingFace’s Transformers library [36]. A sequence-classification head (a linear layer) was added on top of the base model’s hidden states. Radiology reports were then represented as a tokenized sequence according to the requirements of each of the Transformer models–using a punctuation and wordpiece tokenizer (BERT, DistilBERT), SentencePiece tokenizer (XLNet), or Byte-Pair Encoding (RoBERTa). We limited the maximum length of the input sequence to 128, padding shorter sequences with zeros, whilst cropping longer sequences. Subsequently, all models were trained using the Adam optimizer for 20 epochs. Training was terminated early if validation loss did not improve for three consecutive epochs.

### Unsupervised report classification

Reliable ground truths in radiological data is a scarce resource, which requires considerable clinical time and expertise. To address this limitation, we introduce a fully unsupervised approach to assigning Normal and Abnormal labels to free-text radiological reports.

#### Dimensionality reduction using siamese neural networks

The unsupervised ivis algorithm [24] was used to reduce dimensionality of 50-dimensional fastText embeddings of unlabelled reports within the GGC corpus. To obtain report-level embeddings, fastText word vectors within each report were averaged [37] and the resulting 50-dimensional vector was used to as inputs into the ivis algorithm. ivis Siamese Neural Network was initialised using three identical three-layer dense networks consisting of 500, 500, and 2,000 neurons each, followed by an embedding layer with the number of neurons reflecting dimensionality of desired embeddings. The layers preceding the embedding layer use the SELU activation function, which gives the network a self-normalizing property [38]. The weights for these layers are randomly initialized with the LeCun normal distribution. The embedding layers use a linear activation and have their weights initialized using Glorot’s uniform distribution. The network was trained using a triplet loss function, whilst Euclidean distance was used to establish similarity between points in the embedding space [24]. Nearest neighbour selection was limited to 130 points and the training was halted early if the triplet loss did not improve for five epochs.

#### Gaussian mixture model clustering

**Fig 2:**
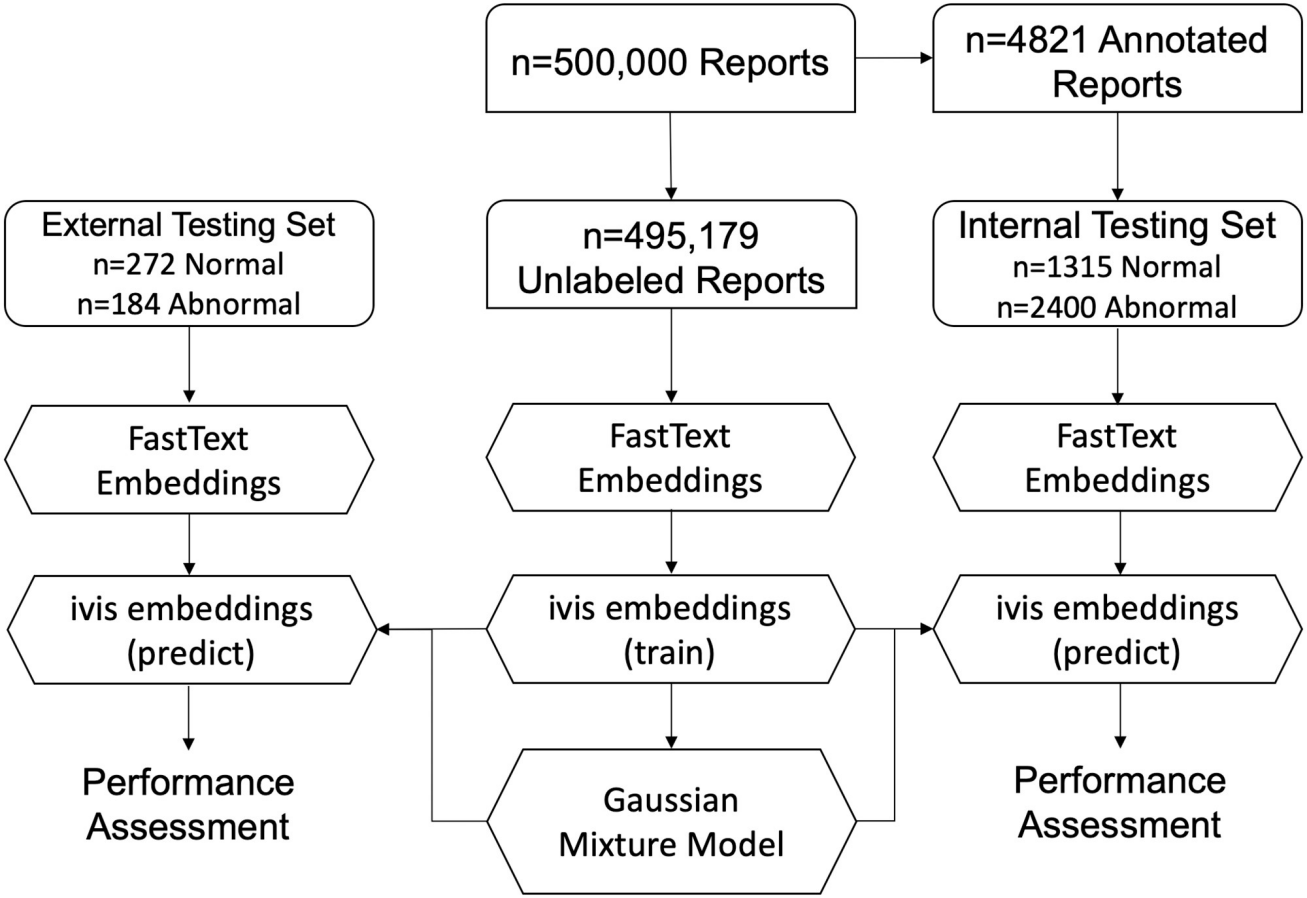
Flowchart demonstrating unsupervised approach to radiology report classification.

A Gaussian Mixture Model (GMM) with two mixture components was applied to either FastText or ivis embeddings (Fig 2). Posterior probabilities of each mixture component were then obtained on the expertly labelled internal (GGC) and external (MIMIC-CXR) testing sets. The GMM’s performance was evaluated by comparing ground truth labels of the testing set to mixture component probabilities.

#### CheXpert labeller

The CheXpert labeller is an NLP tool based on keyword matching with hardcoded rules describing negation [4], which assigns each report with one or more labels associated with thoracic pathology. The labeller operates in three stages: 1) extraction, 2) classification, and 3) aggregation. In the extraction stage, all mentions of a label are identified, including alternate spellings, synonyms, and abbreviations. Mentions are then classified as positive, uncertain, or negative using local context. In cases where keyword matching fails to produce a reliable result, a label of No Findings is assigned. We considered all reports with a label of No Findings to be Normal, whilst remaining reports were considered to be Abnormal.

### Performance assessment

Model performance was assessed on internal (NHS GGC) and external (MIMIC-CXR Database) testing sets. The following performance metrics were recorded–precision, recall, f1-score, and Area Under Receiver Operating Characteristic Curve (AUROC). In multi-class classification problems, we weigh the average of the precision, recall, and f1-score by the number of instances of each class.

## Results

### Supervised report classification

**Fig 3:**
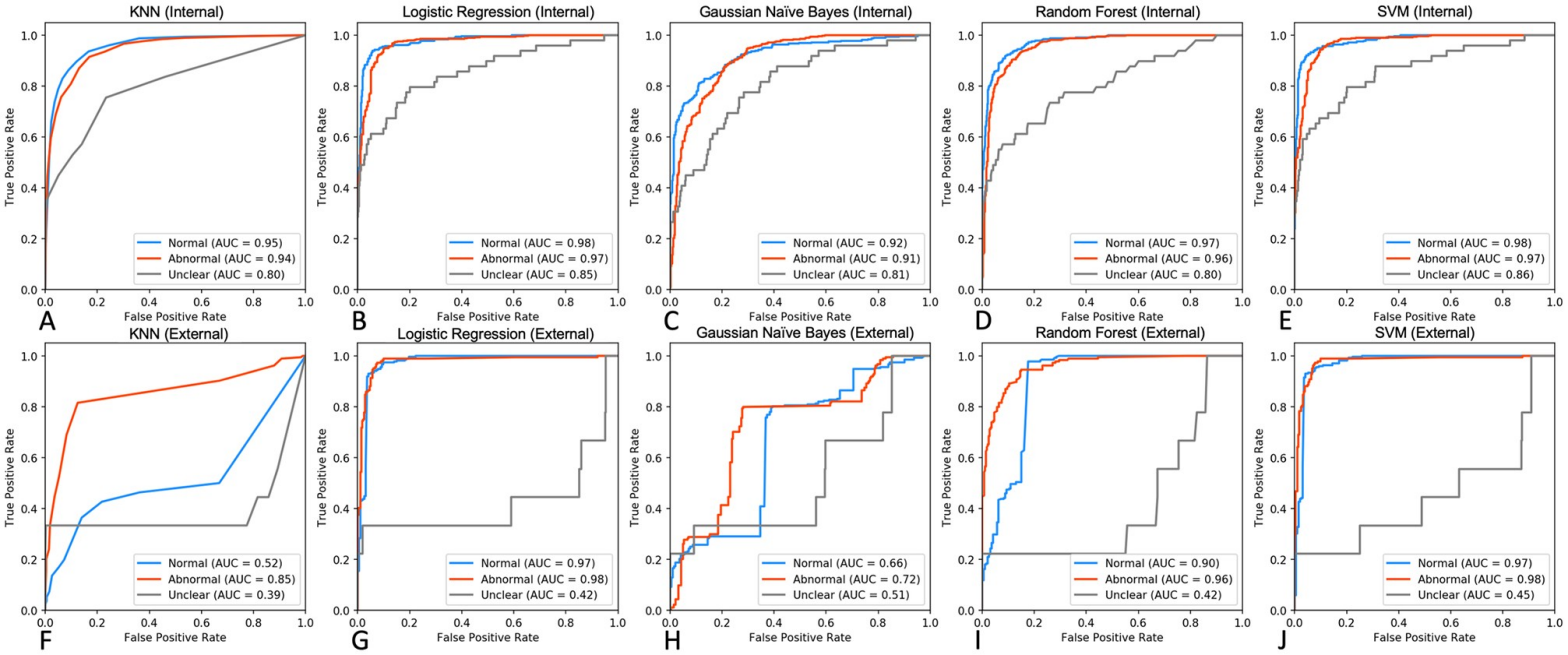
Performance assessment of non-neural classifiers on internal and external testing sets. **A-E.** ROC curves displaying performance metrics on an expert-labelled internal testing set (n = 329 Normal, n = 601 Abnormal, n = 35 Uncertain). **F-G.** ROC curves demonstrating classifier performance on external MIMIC-CXR free-text reports (n = 272 Normal, n = 184 Abnormal, n = 9 Uncertain).

Five supervised multi-class classifiers were trained on tf-idf/SVD-transformed document matrices (n = 1,315 Normal, n = 2,399 Abnormal, n = 142 Uncertain)–KNN, Logistic Regression, Naïve Bayes, Random Forest, and SVM (Fig 3). For each model, an exhaustive grid search was carried out on the training set using 5-fold cross validation, optimising the f1 score, and the best performing parameters were fixed for subsequent performance assessment. SVM and Logistic Regression performed consistently well in identifying Normal and Abnormal reports, both in internal (AUROC 0.97–0.98, Fig 3B and 3E) and external (AUROC 0.97–0.98, Fig 3G and 3J) testing sets. Although SVM performed well in differentiating Unclear reports in the internal testing set (AUC = 0.86), all classifiers yielded suboptimal accuracy for this class in the external set (AUROC 0.39–0.51, Fig 3F–3J).

**Fig 4:**
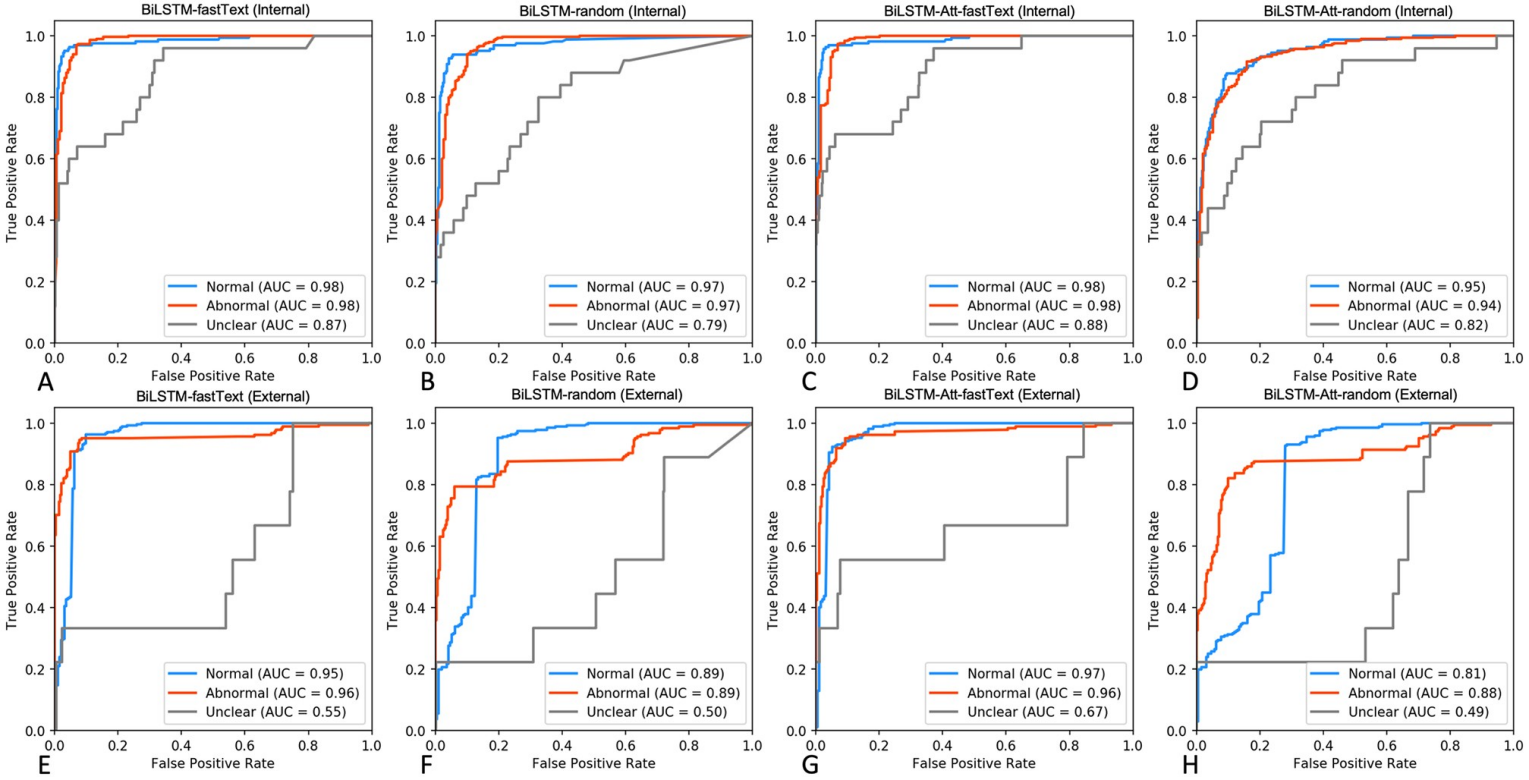
Performance assessment of BiLSTM classifiers on internal and external testing sets. **A-D.** ROC curves displaying performance metrics on an expert-labelled internal testing set (n = 329 Normal, n = 601 Abnormal, n = 35 Uncertain). **E-H.** ROC curves demonstrating classifier performance on the external MIMIC-CXR expert-labelled free-text reports (n = 272 Normal, n = 184 Abnormal, n = 9 Uncertain).

Next, we used the expert-labelled internal radiological reports to train a series of three-class BiLSTM classifiers using tokenised report sequences. We hypothesised that by considering temporal word relationships within each report, a more nuanced and generalisable model could be obtained through modelling radiological language with BiLSTMs. As above, performance was assessed on both internal and external testing sets. Pre-training BiLSTM with fastText embeddings (BiLSTM-fastText) produced robust classifiers compared to randomly initialised model weights (Fig 4A–4D, Table 1). Whilst BiLSTM-fastText resulted in marginally better detection of Unclear class compared to the top-performing SVM classifier (AUC_BiLSTM-fastTet_ = 0.87 vs. AUC_SVM_ = 0.86, Fig 4A), this performance was further superseded by introducing attention mechanism to BiLSTM-fastText architecture (AUC_BiLSTM-Att-fastText_ = 0.88, Fig 4C, Table 1).

**Table 1:** Performance comparison of supervised multi-class classifiers on internal and external testing sets. Class-weighted values are reported.

Classifier	Internal Testing Set (n = 978)			External Testing Set (n = 465)		
	Precision	Recall	F1-score	Precision	Recall	f1-score
*KNN*	0.82	0.86	0.84	0.81	0.82	0.81
*Logistic Regression*	0.85	0.90	0.87	0.91	0.93	0.92
*Naïve Bayes*	0.78	0.82	0.80	0.70	0.47	0.38
*Random Forest*	0.83	0.88	0.86	0.84	0.80	0.80
*SVM*	0.85	0.90	0.88	0.91	0.93	0.92
*BiLSTM-fastText*	0.93	0.93	0.93	0.91	0.91	0.91
*BiLSTM-random*	0.91	0.91	0.91	0.72	0.58	0.57
*BiLSTM-Att-fastText*	**0.94**	**0.94**	**0.94**	0.90	0.91	0.90
*BiLSTM-Att-random*	0.90	0.91	0.90	0.73	0.55	0.53
*BERT*	0.92	0.93	0.92	0.94	0.93	0.93
*DistilBERT*	0.91	0.91	0.91	0.93	0.93	0.93
*XLNet*	0.93	0.93	0.93	**0.95**	**0.95**	**0.95**
*RoBERTa*	0.91	0.91	0.91	0.93	0.93	0.93

To assess how well our BiLSTM models generalise to an external testing set, we compared predicted labels to manually annotated reports from the MIMIC-CXR database (n = 272 Normal, n = 184 Abnormal, n = 9 Unclear, Fig 4E and 4F). Randomly-initialised BiLSTMs (BiLSTM-random) exhibited worse performance compared to both fastText-pretrained models and non-neural classifiers (Fig 4F and 4H). Interestingly, BiLSTM-Att-fastText, generalised well across Normal and Abnormal classes, and performed favourably on the Unclear class in the internal and external testing sets (Fig 4C and 4G).

**Fig 5:**
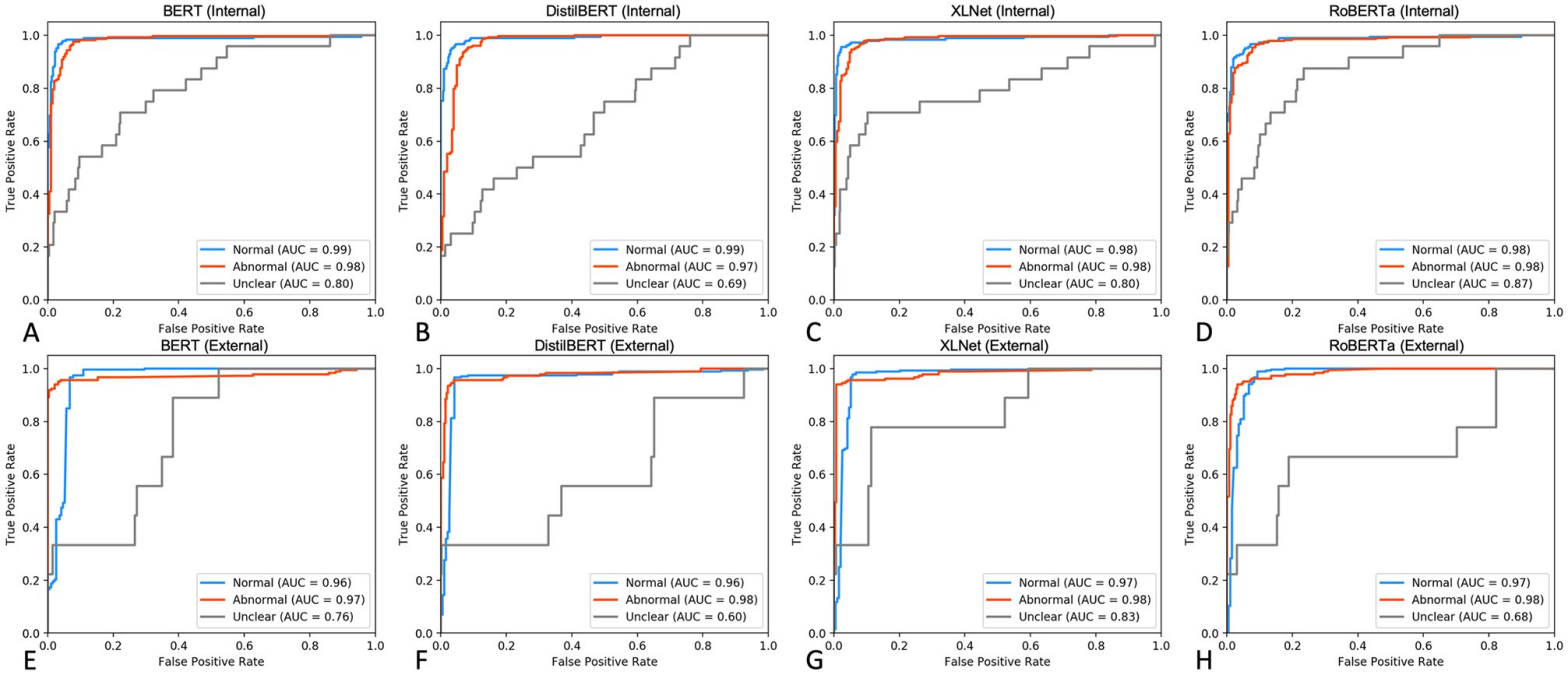
Performance assessment of Transformer-based classifiers on internal and external testing sets. **A-D.** ROC curves displaying performance metrics on an expert-labelled internal testing set (n = 329 Normal, n = 601 Abnormal, n = 35 Uncertain). **E-H.** ROC curves demonstrating classifier performance on the external MIMIC-CXR expert-labelled free-text reports (n = 272 Normal, n = 184 Abnormal, n = 9 Uncertain).

Finally, four self-attention based models (BERT, DistilBERT, XLNet, and RoBERTa) were evaluated on the internal and external testing sets (Fig 5). Whilst all models performed well in classifying Normal and Abnormal reports (AUC = 0.97–0.99), XLNet achieved favourable performance on the Unclear class in both internal and external testing sets (AUC = 0.80 and AUC = 0.83 respectively, Fig 5C and 5G).

### Unsupervised report classification

We demonstrated that supervised classifiers achieve excellent performance using a relatively small number of labelled training exemplars (n = 3,856 reports). Furthermore, neural networks that utilise the BiLSTM architecture with attention mechanism appear to generalise well to external radiological reports. Nevertheless, generation of reliable ground truths remains a barrier to training effective deep learning models due to the required clinical time and expertise. To address this limitation, we set out to develop and evaluate an unsupervised approach to assigning Normal and Abnormal labels to free-text radiological reports.

**Fig 6:**
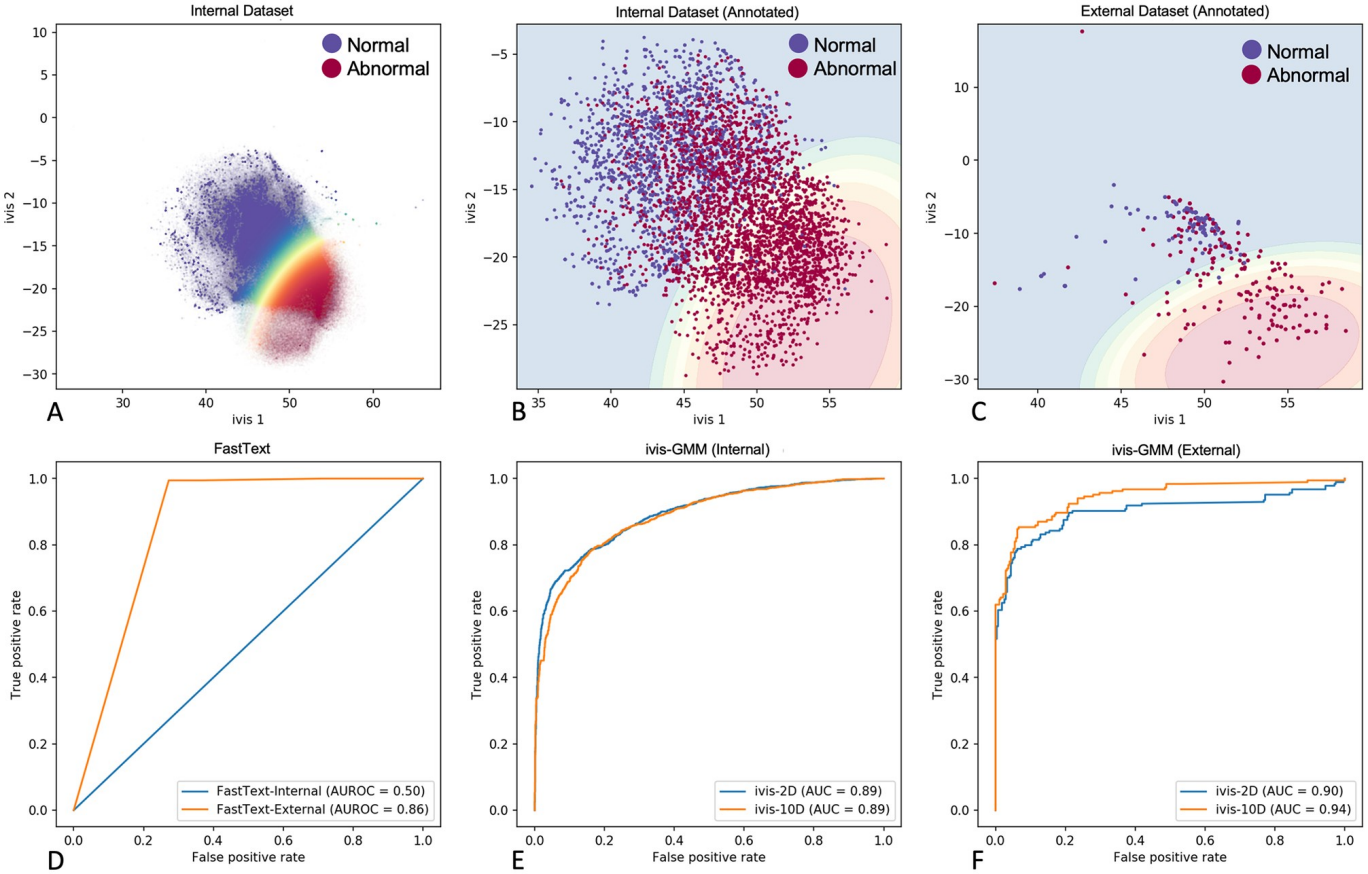
Unsupervised report classification using fastText, ivis, and Gaussian Mixture Model clustering. **A.** Two-dimensional ivis representation of 50-dimensional fastText embeddings of n = 495,179 unlabelled radiological reports from NHS GGC. Colour gradient reflects posterior probability of Normal and Abnormal report cluster. **B.** Scatterplot of predicted ivis embeddings for n = 3,715 expert-labelled reports in the internal testing set. Blue and red points represent manually-labelled Normal and Abnormal reports respectively. Colour gradient reflects contours of posterior probability distributions obtained from GMM model trained on two-dimensional ivis representations of n = 495,179 unlabelled radiological reports. **C.** Scatterplot of predicted ivis embeddings for n = 456 expert-labelled reports in the MIMIC-CXR testing set. Blue and red points represent manually-labelled Normal and Abnormal reports respectively. Colour gradient reflects contours of posterior probability distributions obtained from GMM model trained on two-dimensional ivis representations of n = 495,179 unlabelled radiological reports. **D**. ROC curves of unsupervised GMM classifier applied to 50-dimensional fastText embeddings of internal (n = 3,715) and external (n = 456) manually-labelled reports. **E-F**. ROC curves of unsupervised GMM classifier applied to two- and ten-dimensional ivis embeddings of manually labelled internal (n = 3,715) and external (n = 456) reports.

An internal corpus of n = 495,179 unlabelled reports was represented as a collection of 50-dimensional fastText document vectors (see Methods). We hypothesised that free-text entities can be modelled using Gaussian Mixture Distributions due to inherently distinct semantic structure of Normal and Abnormal reports. To test this hypothesis, a GMM with two components was constructed from the unlabelled document vectors and posterior probabilities of each component were extracted from the expert-labelled reports of the internal (n = 1,315 Normal and n = 2,400 Abnormal) and external (MIMIC-CXR, n = 272 Normal and n = 184 Abnormal) corpora. Whilst GMM performance on an internal testing set was sub-optimal, model validation on an external testing set produced acceptable metrics (AUC = 0.50 and AUC = 0.86 respectively, Fig 6D).

Recently, we introduced a novel algorithm, ivis, for dimensionality reduction and feature engineering in large datasets [14]. ivis is a parametric method that utilises a Siamese Neural Network to generate low-dimensional data representations that preserve both local and global properties of original observations. To further refine fastText embeddings, we applied ivis to 50-dimensional report vectors prior to GMM clustering (Fig 6A). Reduction of fastText reports to two-dimensional ivis representations resulted in marked performance improvements in both internal and external datasets (AUC = 0.89 and AUC = 0.90 respectively, Fig 6B and 6C). GMM performance was enriched further by expanding ivis representations to ten embedding dimensions (ivis-10D, AUC_Internal_ = 0.89, AUC_External_ = 0.94, Fig 6E and 6F).

Finally, we compared GMM-clustered ivis embeddings to annotations generated by the CheXpert Labeller. The labeller is a rule-based classifier which operates in three stages: 1) extraction, 2) classification, and 3) aggregation. In the extraction stage, all mentions of a label are identified, including alternate spellings, synonyms, and abbreviations. Mentions are then classified as positive, uncertain, or negative using local context. The CheXpert Labeller is tailored for CXRs, and recently demonstrated favourable performance on free-text reports [23]. Both the Labeller and GMM-clustered ivis-10D embeddings achieved comparable performances on an internal and external dataset (f1-score_Internal_ = 0.81 and f1-score_External_ = 0.92, Table 2). Interestingly, just two-dimensional ivis embeddings achieved acceptable classification performance, making the datasets amenable to interpretable visualisation (Fig 6A–6C).

**Table 2:** Performance comparison of unsupervised classifiers on internal and external radiological reports. Average performance values are reported.

Classifier	Internal Testing Set (n = 3715)			External Testing Set (n = 456)		
	Precision	Recall	f1-score	Precision	Recall	f1-score
*fastText+GMM*	0.42	0.65	0.51	0.88	0.84	0.84
*ivis-2D+GMM*	**0.83**	0.80	0.80	0.88	0.88	0.88
*ivis-10D+GMM*	0.82	0.80	**0.81**	0.91	0.91	**0.92**
*CheXpert Labeller*	0.81	**0.81**	**0.81**	**0.93**	**0.93**	**0.92**

## Discussion

In this work we examine the application of supervised machine learning algorithms to classification of free-text CXR reports. Rigorous performance benchmarking on two independent corpora from two international health systems demonstrate that BiLSTM networks with self-attention mechanism produce state-of-the-art classification results and are generalise to external testing sets. Furthermore, we introduce a fully unsupervised approach for abnormality detection in free-text reports, which performs favourably compared to a well-established rules-based classifier tuned for CXR labelling.

Our analysis of five non-neural supervised classifiers (KNN, Logistic Regression, Naïve Bayes, Random Forest, and SVM) demonstrated that whilst all models achieved excellent performance on an internal testing set, only SVM successfully captured Normal and Abnormal entities in both testing sets. Reports labelled as Unclear were consistently misclassified by all algorithms in the external testing set (Fig 3F–3J). These findings are consistent with the general notion that SVMs are well-suited for a text classification task due to 1) the algorithm’s ability to learn independently of the dimensionality of the feature space, 2) suitability to problems with dense concepts and sparse instances (document vectors are sparse as each document vector contains only few entries which are not zero), and 3) linearly separable nature of most text problems [39]. Indeed, an SVM trained on a bag of phrases was used to detect hospital admissions due to specific diseases [40] as well as classify medical subdomain across clinical notes [41]. Interestingly, the SVM only marginally outperforms Logistic Regression classifier. Considering that Logistic Regression predictions may be viewed as locally interpretable [42], the minor trade-off in accuracy may be justified in favour of trusting and understanding intuition behind each classification [43].

Non-neural classifiers rely on a bag-of-words (BOW) representation of the training corpus. The approach maintains word multiplicity, but disregards grammatical nuances and word order of original sentences. Additionally, BOW matrices are often sparse, with only a few entries which are not zero. This convention has often proven to be problematic for non-neural classifiers due to the data sparsity problem [44]. In recent years, deep artificial neural networks have been found to yield consistently good and often state-of-the-art results on a variety of NLP tasks [18]. It can be argued that by considering complex inter-relationships between words within sentences, deep neural networks achieve state-of-the-art performance across NLP tasks such as part-of-speech tagging, shallow parsing, named entity recognition, and semantic role labelling [45].

We demonstrated that BiLSTM networks learn to differentiate Normal and Abnormal CXR reports and generalise well to an independent testing set (Table 1). Traditionally, BiLSTMs have shown performance improvements in NLP tasks over Unidirectional LSTMs, lkely due to inclusion of information from both future and past words in the sentence [28]. We demonstrate that an important requirement to a generalisable model is initialising the network with pre-trained word embeddings. Indeed, pre-trained BiLSTMs weights considerably outperformed random weight initialisation in terms of precision, recall, and f1-scores (Table 1). Previous reports have shown only marginal accuracy gains attributed to pre-training [18]. However, this is like because only an internal testing set was used to benchmark algorithm performance, whilst we note considerable gains on external datasets.

To pre-train our models, we applied fastText to an unlabelled corpus of n = 495,179 reports from NHS GGC. Several important features prompted us to choose fastText over other comparable approaches. First, the algorithm is fast and can train on our corpus within a few minutes. This allowed us to experiment with hyperparameters in order to produce better embeddings. Second, fastText operates at a character level, meaning that word vectors can still be extracted for those words that are not present in the original vocabulary. This is especially important as spelling and abbreviations vary greatly between radiological reports [46]. Indeed, it is not unreasonable to hypothesise that this feature of the fastText algorithm contributed significantly to model generalisability across testing sets. Finally, unlike word vectors from word2vec [47], fastText word features can be averaged together to form good sentence representations [37]. It is plausible that adding more reports into the pre-training corpus will lead to further performance gains–this is something that we intend to explore in greater detail as our work evolves.

In an earlier work, the attention mechanism was demonstrated to achieve high accuracy on an expertly labelled CXR dataset (f1-score = 0.93) [19]. The attention layer learns heterogeneous text representations for each label under an assumption that each snippet containing distinguishing information could be anywhere in the text and would differ across labels [19]. As such, attention can help combine global and local information in order to improve classification performance [48]. In this work, a BiLSTM (pre-trained with fastText vectors) with attention mechanism was the top-performing LSTM classifier (f1-score_Internal_ = 0.94, f1-score_External_ = 0.90, Table 1). Interestingly, the approach also identified Uncertain reports considerably better on an external testing set (AUC = 0.67, Fig 4G), suggesting that more nuanced information can still be learnt from small number of exemplars. Overall, this work, together with independent reports [19, 48, 49], suggests that training a BiLSTM with attention on a relatively small corpus of labelled data produces generalisable state-of-the-art free-text classifiers that may augment training of computer vision models.

Recently, several novel network architectures, based solely on attention mechanisms, have achieved state-of-the-art performance across NLP tasks [31]. In this work we demonstrate that four Transformer-based models, namely BERT, DistilBERT, XLNet, and RoBERTa, achieve excellent performance (AUC: 0.97–0.99, Fig 5) on free-text radiological reports, which generalises well to an external testing set. Although performances of all Transformer-based models were comparable to BiLSTM with attention mechanism for Normal and Abnormal reports, XLNet identified Uncertain reports with increased accuracy (AUC_XLNet_ = 0.80–0.83 vs. AUC_BiLSTM-Att-fastText_ = 0.67–0.88). This improvement is likely due to the capacity of XLNet to learn bidirectional contexts and its autoregressive formulation [34]. Nevertheless, despite marginal increase in performance, training and finetuning Transformer-based models is a computationally expensive task. Marginal performance gains are offset by the hardware resources required to complete training and inference experiments.

So far, we have shown that supervised classifiers achieve excellent performance using a relatively small number of labelled training exemplars (n = 3,856 reports). Nevertheless, generation of reliable ground truths remains a barrier to training effective deep learning models due to the required clinical time and expertise [11]. To address this limitation, we set out to develop and evaluate an unsupervised approach to assigning Normal and Abnormal labels to free-text radiological reports. Our top-performing approach involves three steps: 1) obtaining sentence vectors by averaging fastText word features, 2) feature extraction using ivis, a novel Siamese Network algorithm, and 3) fitting a GMM with two components (assuming that distributions of Normal and Abnormal reports are inherently different) to ivis embeddings. Performance of this three-step approach was comparable to the CheXpert Labeller, which utilises hand-crafted rules tailored for CXR report annotation [4, 23]. We have previously applied ivis to structured single-cell datasets [24], demonstrating that the algorithm reliably preserves local and global distances in a low-dimensional space. Briefly, ivis employs a Siamese Neural Network architecture that learns to discriminate between similar and dissimilar fastText vectors without imposing strong priors. This property enables natural creation of dense clusters with shared nearest neighbours, making sentence vectors amenable to modelling with GMMs. Interestingly, although ivis was trained on the unlabelled internal corpus, it performed considerably better on an external testing set (Table 2). This was also the case for the CheXpert Labeller. It is likely that given that external testing set reports were shorter than internal reports (external average: 13.4 words vs. internal average: 33.2 words), the external reports were more linearly separable (Fig 6C), resulting in improved unsupervised performance.

Whilst ivis+GMM performance was comparable to CheXpert Labeller, we have identified several advantages of our unsupervised approach. First, GMMs can be used to obtain posterior probabilities of each component for every report. This provides a degree of granularity to our results. For example, at a component probability threshold greater than 0.99, ivis+GMM identified 30% of Abnormal reports with 100% positive predictive value. Conversely, the CheXpert Labeller provides strictly categorical outputs, that cannot be used to fine-tune an algorithm’s confidence. Second, ivis+GMM is a general approach and is not restricted to CXR reports. It is likely that application of this algorithm will yield comparable results in other free-text medical records. Finally, ivis is a dimensionality reduction technique, which can be used to visualise complex data structures in two-dimensional space. It has been shown to scale linearly to millions of data points, resulting in more interpretable visualisations than comparable techniques such as t-distributed Stochastic Neighbour Embedding (t-SNE) [50].

Taken together, we have shown that supervised machine learning algorithms can reliably label free-text CXR radiological reports with excellent performance and using a relatively small number of training exemplars. More specifically, pre-training BiLSTM with fastText weights and the inclusion of the attention mechanism yields state-of-the-art accuracy that can be generalised to an independent testing set. To the best of our knowledge this is the first study where the generalisability of a machine learning algorithm for free-text CXR report interpretation has been demonstrated across two independently sourced and expert-labelled testing sets. Furthermore, we validate a general fully unsupervised approach that utilises Siamese Neural Networks and GMMs to reliably label large free-text corpora. Although direct application of expert knowledge to unlabelled radiograms remains the gold-standard of image annotation, we anticipate that our results can be used to effectively extract standardized clinical information from CXR radiological reports, facilitating large-scale training of modern clinical decision support systems for CXR triage.
